# Network pharmacology-based pharmacological mechanism prediction of Lycii Fructus against postmenopausal osteoporosis

**DOI:** 10.1097/MD.0000000000036292

**Published:** 2023-12-01

**Authors:** Jianbo Wang, Yi Wang, Leyan Li, Shuiqi Cai, Dandan Mao, Hongkan Lou, Jian Zhao

**Affiliations:** a Department of Orthopedic Surgery, The Third People’s Hospital Health Care Group of Cixi, Ningbo, China; b Department of Oncology, Ningbo Municipal Hospital of Traditional Chinese Medicine, Ningbo, China; c The 3rd School of Clinical Medicine, Zhejiang Chinese Medicine University, Hangzhou, China; d Department of Orthopedic Surgery, Ningbo Municipal Hospital of Traditional Chinese Medicine, Ningbo, China; e Changzhou No.2 People’s Hospital Affiliated to Nanjing Medical University, Changzhou, China.

**Keywords:** Lycii Fructus, network pharmacology, postmenopausal osteoporosis

## Abstract

Postmenopausal osteoporosis (PMOP) has become one of most frequent bone diseases worldwide with aging population. Lycii Fructus, a common plant fruit with the property of drug homologous food, has long since been used to treat PMOP. The aim of this study is to explore pharmacological mechanisms of Lycii Fructus against PMOP through using network pharmacology approach. The active ingredients of Lycii Fructus were obtained from Traditional Chinese Medicine System Pharmacology database. Target fishing was performed on these ingredients in UniProt database for identification of the relative targets. Then, we screened the targets related to PMOP using GeneCards database and DisGeNET database. The overlapping genes between PMOP and Lycii Fructus were obtained to perform protein–protein interaction, gene ontology analysis, Kyoto Encyclopedia of Genes and Genomes analysis. A total of 35 active ingredients were identified in Lycii Fructus, and fished 158 related targets. Simultaneously, 292 targets associated with PMOP were obtained from GeneCards database and DisGeNET database. By drawing Venn diagram, 41 overlapping genes were obtained, and were considered as therapeutically relevant. Gene ontology enrichment analysis predicted that anti-inflammation and promotion of angiogenesis might be 2 potential mechanism of Lycii Fructus for PMOP treatment. Kyoto Encyclopedia of Genes and Genomes enrichment analysis revealed several pathways, such as IL-17 pathway, TNF pathway, MAPK pathway, PI3K-Akt signaling pathway and HIF signaling pathway were involved in regulating these 2 biological processes. Through the method of network pharmacology, we systematically investigated the mechanisms of Lycii Fructus against PMOP. The identified multi-targets and multi-pathways provide new insights to further determinate its exact pharmacological mechanisms.

## 1. Introduction

Postmenopausal osteoporosis (PMOP) is a common chronic bone disease that majorly occurred in women over 50 years old.^[1]^ PMOP presents bone mass loss and microstructure deterioration, which apes to cause patients’ low back pain, hunchback and fragility fractures.^[2]^ It is estimated that about 15% of postmenopausal women are suffering in PMOP worldwide,^[3]^ and the percentage will be continually raising with aging population. Currently, the treatment strategies for PMOP are consist of calcium, bone formation promoters (e.g., fluorides, parathyroid hormone) and bone resorption inhibitors (e.g., bisphosphonates, calcitonin and estrogen receptor modulators).^[4,5]^ Although these drugs can alleviate bone loss and other symptoms, various adverse effects limit their widely clinical application.^[6,7]^ Therefore, exploring safer and more effective therapeutic treatments are largely needed. Due to relative safety, natural products and herbs grab increasing attention to develop new anti-osteoporosis drugs.^[8]^

Lycii Fructus also called Gouqizi is the fruit of plant Lycium barbarum, and was firstly described in Sheng Nong’s herbal classic as a Chinese kidney-tonifying herb.^[9]^ Also, Lycii Fructus is widely used in home cooking such as tea, soups and porridge for its Chinese Pharmacopeia and pleasant flavor.^[10]^ Lycii Fructus is used alone or mixed with other herbs to treat bone diseases for a long history.^[11-13]^ Previous studies have demonstrated that Lycii Fructus has a bone protect effect and inhibits bone loss in ovariectomized (OVX) mice.^[13,14]^ This anti-osteoporosis effect can be well explained in traditional Chinese medicine theory of “kidney governing bone,” that PMOP occurs due to kidney deficiency, and Lycii Fructus strengths bone through nourishing kidney.^[15,16]^ Modern pharmacological composition researches revealed that Lycii Fructus was rich in physiological bioactive properties such as kaempferol, quercetin, oleanolic acid, dihydrophaseic acid and urosolic acid.^[17]^ Nevertheless, it remains largely unclear that the pharmacological mechanisms of Lycii Fructus against PMOP.

As multi-components and multi-targets involved in therapeutic mechanisms of Chinese herbs, traditional “one drug, one target” study strategy can not meet the requirements to analyze such numerous compounds and targets simultaneously.^[18–20]^ Network pharmacology integrates pharmacology, biology, bioinformatics and computer science, and is an ideal method to comprehensively study muti-components and muti-targets as a whole.^[21,22]^ With the aid of database information and network construction of “Medicine-Targets-pathways-Disease,” network pharmacology can effectively predict the potential mechanisms of Chinese herbs in treating various diseases.^[23–25]^

**Figure 1.**
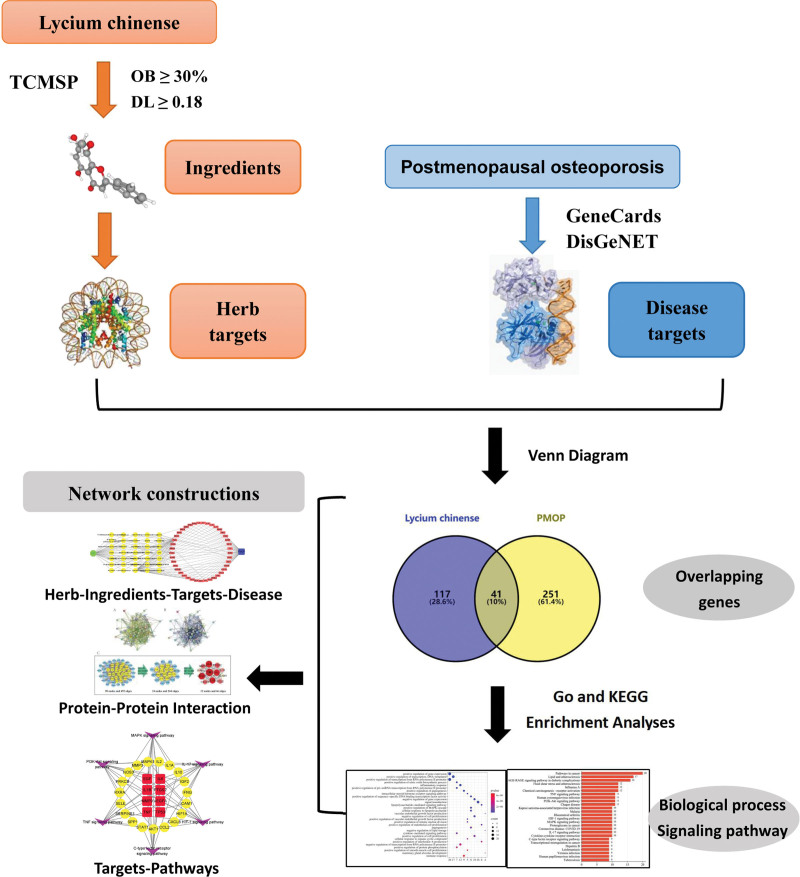
The flow chart of network pharmacology-based strategy for investigating potential pharmacological mechanism of Lycii Fructus against PMOP. PMOP = postmenopausal osteoporosis.

In the present study, we investigated the pharmacological mechanisms of Lycii Fructus against PMOP by using network pharmacology. The targets related to Lycii Fructus and PMOP were screened out through several databases. Based on their overlapping target genes, we built a protein–protein interaction (PPI) network to analyze their interactions, and performed gene ontology (GO) and Kyoto Encyclopedia of Genes and Genomes (KEGG) analyses to predict the key biological processes and signaling pathways. The specific protocol was shown in Figure 1.

## 2. Material and methods

As all procedures performed in the present study did not involve human participants or animals, ethical approval was not necessary.

### 2.1. Collection and target prediction of active ingredients of Lycii Fructus

“Lycii Fructus” or “Qouqizi” was inputted into Traditional Chinese Medicine System Pharmacology Database (TCMSP, http://lsp.nwu.edu.cn/tcmsp.php, Version 2.3) to collect the ingredients of Lycii Fructus. Oral bioavailability (OB) is defined as the efficiency of drug delivery to the systemic circulation. Drug-likeness (DL) is a qualitative concept used to assess drug properties including solubility and chemical stability. Here, OB ≥ 30% and DL ≥ 0.18 were set as the screening filters to identify the active ingredients of Lycii Fructus.^[26]^ Then, we obtained the active ingredient-related targets by using the computer targeting technology contained in the TCMSP, and further transformed the target name to a standard gene symbols in the Uniport database (https://www.uniprot.org/) with the filters of “swiss-prot reviewed” and “Homo sapiens.”

### 2.2. PMOP-related targets

DisGeNET database (https://www.disgenet.org/) and GeneCards database (https://www.genecards.org/) were used to select targets associated with PMOP. Through inputting “postmenopausal osteoporosis” and further screening with the filters of score > 0.1 in DisGeNET and score > 10 in GeneCards,^[26]^ a total of 292 PMOP-related targets were finally obtained.

### 2.3. Venn diagram

A Venn diagram was constructed on an online website (https://bioinfogp.cnb.csic.es/tools/venny/index.html) to identify the overlapping target genes between Lycii Fructus and PMOP for further bioinformatic analyses.

### 2.4. PPI network

With the parameters of “confidence score > 0.4” and “Homo sapiens,” 41 overlapping genes between Lycii Fructus and PMOP were analyzed in STRING (https://string-db.org/) to build a PPI network. Then, the gene-gene connection data was exported from STRING database, and further analyzed their interconnection network by using the visualized software Cytoscape 3.7.1. Three topological parameters including degree centrality (DC), betweenness centrality (BC), and closeness centrality (CC) were used to evaluate the hub target genes.

### 2.5. GO and KEGG enrichment analysis

The overlapping genes between Lycii Fructus and PMOP were inputted into the Annotation, Visualization and Integrated Discovery (https://david.ncifcrf.gov/home.jsp, version 6.8) for GO enrichment analysis. Three sub-items including molecular function, cellular component (CC) and biological process (BP) were all analyzed. After filtered by FDR > 0.5, the information of top 30 BP terms were listed in a bubble diagram based on the ascending order of log *P*-value. KEGG enrichment analysis was performed on the overlapping genes by inputting them into the KEGG (https://www.kegg.jp/) database. Based on the descending order of gene number enriched in each pathway, we listed the top 26 signaling pathways in a bar diagram.

### 2.6. Network construction

The Cytoscape 3.7.1 software was used to establish the visualized graphs of networks including a Herb-Ingredient-Target-Disease network and a Target-Pathway network

## 3. Results

### 3.1. Prediction of active ingredients and target genes of Lycii Fructus

By searching “Lycii Fructus” or “Gouqizi” in the TCMSP database with the thresholds of OB ≧ 30% and DL ≧ 0.18, a total of 35 active ingredients were identified in Lycii Fructus. Then, these ingredients were used to fish the related targets through TCMSP prediction system. After inputting the related targets into the Uniprot database, a total of 158 target genes of Lycii Fructus were finally obtained.

### 3.2. Overlapping target genes between Lycii Fructus and PMOP

**Figure 2.**
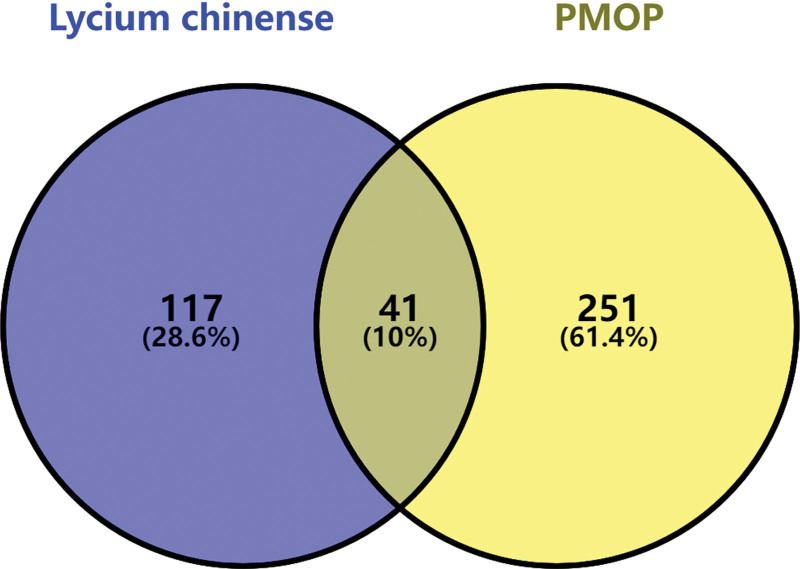
The Venn diagram identified the overlapping targets between Lycii Fructus and PMOP. PMOP = postmenopausal osteoporosis.

**Figure 3.**
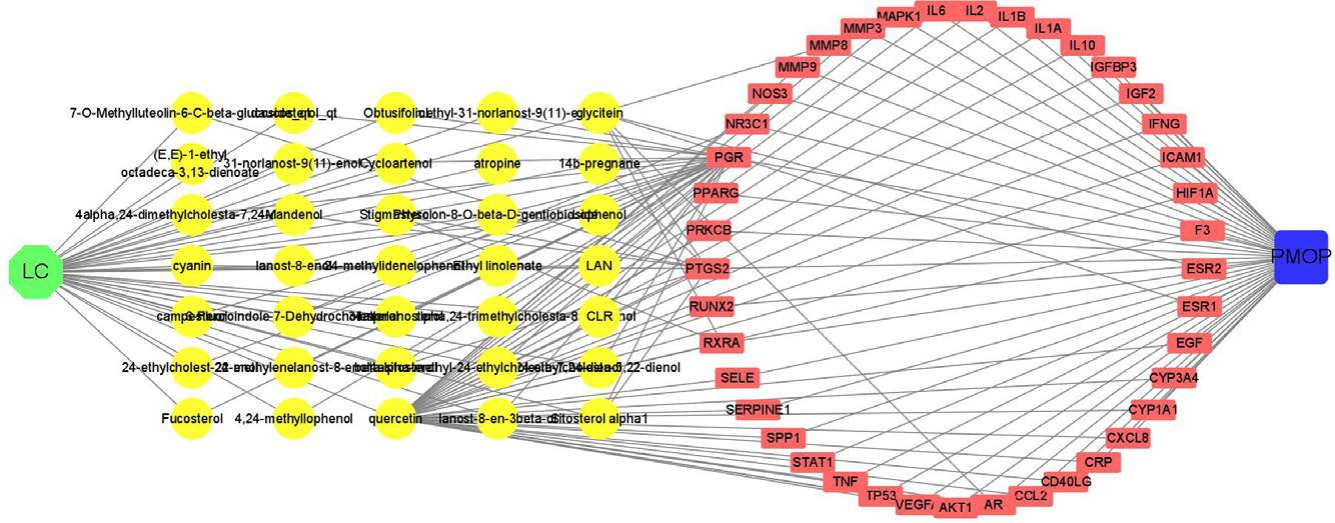
A Herb-Ingredient-Target-Disease network construction. The green hexagon node represents Lycii Fructus, the yellow round nodes represent the ingredients, the pink oblong nodes represent gene targets and the blue quadrate node represents PMOP. PMOP = postmenopausal osteoporosis.

Meanwhile, we searched “postmenopausal osteoporosis” in the DisGeNet database and the GeneCards database with the thresholds of given score, and obtained 292 target genes. Through collecting the co-part between Lycii Fructus and PMOP in the Venn diagram (Fig. 2), we identified 41 overlapping genes that were regarded as the therapeutic targets of Lycii Fructus treating PMOP. Furthermore, a Herb-Ingredient-Target-Disease network was constructed (Fig. 3). According to the number of edge connected target nodes with ingredient nodes, we identified the top 3 ingredients including quercetin (MOL000098, OB = 46.43, DL = 0.28), glycitein (MOL008400, OB = 50.48, DL = 0.28), stigmasterol (MOL000449, OB = 43.83, DL = 0.76). These 3 ingredients connected with 36, 6 and 3 overlapping genes respectively, might be the material foundation for its anti-PMOP effects.

### 3.3. PPI networks and hub therapeutic targets

**Figure 4.**
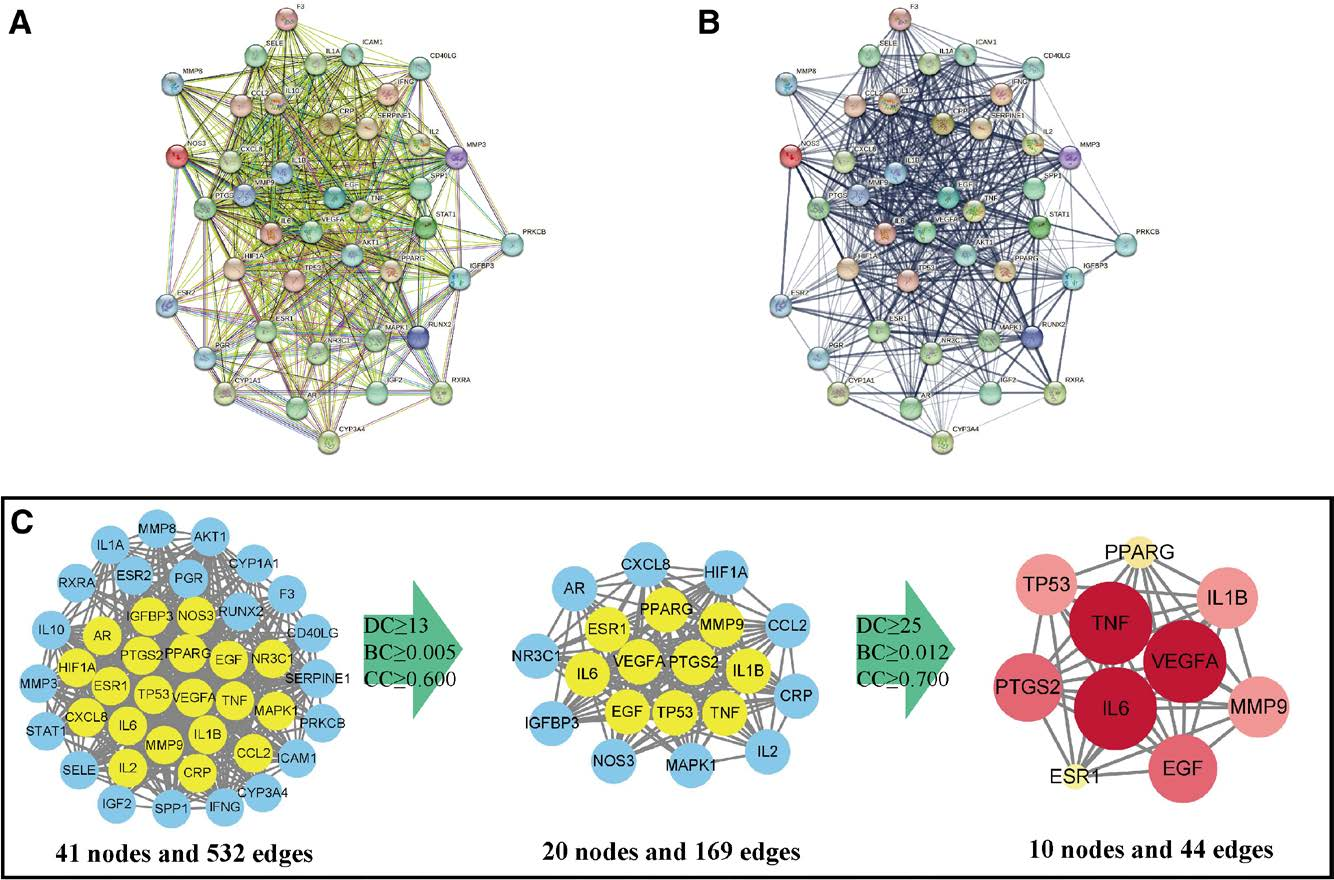
Construction and screening of PPI network. (A) PPI networks constructed in String database by using 41 overlapping targets. Line colors indicate the type of interactive evidence. Line thickness indicates the confidence level of the supporting data. (B) The topological screening with DC, BC, and CC on PPI network. In the third image, the node with higher DC value represents more brilliant color and bigger size. BC = betweenness centrality, CC = closeness centrality, DC = degree centrality, PPI = protein–protein interaction.

Next, these 41 overlapping genes were used to establish a PPI network by using the String database. According to the strength of evidence level or confidence level, we constructed a PPI networks with 41 nodes and 532 edges, respectively (Fig. 4A and B). More line colors indicated higher interactive evidence between 2 nodes (Fig. 4A), and more thickness line reflected stronger confidence level of the supporting data (Fig. 4B). Moreover, 3 parameters including DC, BC and CC were used to select the pivot nodes. Through the first screening round with the thresholds of DC ≥ 13, BC ≥ 0.005 and CC ≥ 0.600, we obtained 169 edges and 20 nodes. After screening with the thresholds of DC ≥ 25, BC ≥ 0.012 and CC ≥ 0.700, only 44 edges and 10 nodes were left (Fig. 4C). Therefore, these remained 10 genes including VEGFA, TNF, IL-6, PTGS2, EGF, TP53, MMP9, IL1B, PPARG and ESR1 were considered as the hub therapeutic targets of Lycii Fructus against PMOP. Their specific information were listed in Table 1.

**Table 1:** Information of 10 hub targets.

Uniprot ID	Gene symbol	Protein name	Degree
P15692	VEGFA	Vascular endothelial growth factor A	38
P01375	TNF	Tumor necrosis factor	38
P05231	IL-6	Interleukin-6	38
P35354	PTGS2	Prostaglandin G/H synthase 2	36
P01133	EGF	Pro-epidermal growth factor	36
P04637	TP53	Cellular tumor antigen p53	35
P14780	MMP9	Matrix metalloproteinase-9	35
P01584	IL1B	Interleukin-1 beta	35
P37231	PPARG	Peroxisome proliferator-activated receptor gamma	31
P03372	ESR1	Estrogen receptor	30

### 3.4. Biological processes of Lycii Fructus in treating PMOP

**Figure 5.**
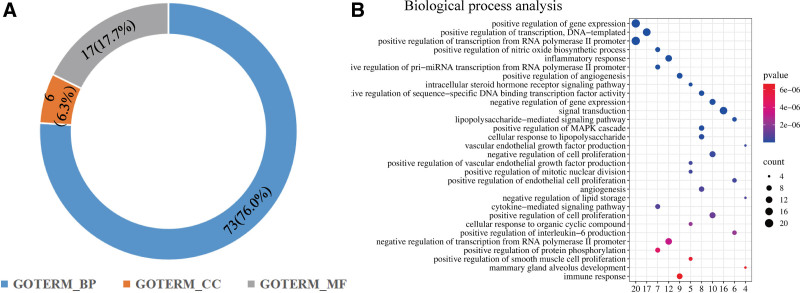
GO enrichment analysis on 41 overlapping genes. (A) The percentage of Three types of GO items in DAVID database (GOTRIM_BP: biological process; GOTRIM_MF: molecular function; GOTRIM_CC: cellular component). (B) The bubble diagram of the top 30 BP items according to descending order of *P* value. GO = Gene Ontology.

The 41 overlapping genes were inputted into the Annotation, Visualization and Integrated Discovery database for GO enrichment analysis. After screening GO items with the thresholds of FDR < 0.05, a total of 96 GO items were identified, and BP subset items occupied a major part (Fig. 5A). Then, we build a bubble diagram to list the contents of top 30 BP terms (Fig. 5B). As the diagram shown, these 30 BP terms could be summarized into 2 category including inflammation and angiogenesis. In the aspect of inflammation, inflammatory response (GO:0006954), intracellular steroid hormone receptor signaling pathway (GO:0030518), lipopolysaccharide-mediated signaling pathway (GO:0031663), cellular response to lipopolysaccharide (GO:0071222), cytokine-mediated signaling pathway (GO:0019221), positive regulation of interleukin-6 production (GO:0032755) and immune response (GO:0006955). In the category of angiogenesis, positive regulation of angiogenesis (GO:0045766), vascular endothelial growth factor production (GO:0010573), negative regulation of cell proliferation (GO:0008285), positive regulation of endothelial cell proliferation (GO:0001938), positive regulation of vascular endothelial growth factor production (GO:0010575), positive regulation of endothelial cell proliferation (GO:0001938) and angiogenesis (GO:0001525). These findings indicated that Fructus Lycii exacts therapeutic effects mainly through 2 biological processes, inflammation and angiogenesis.

### 3.5. Signaling pathways of Fructus Lycii for PMOP treatment

**Figure 6.**
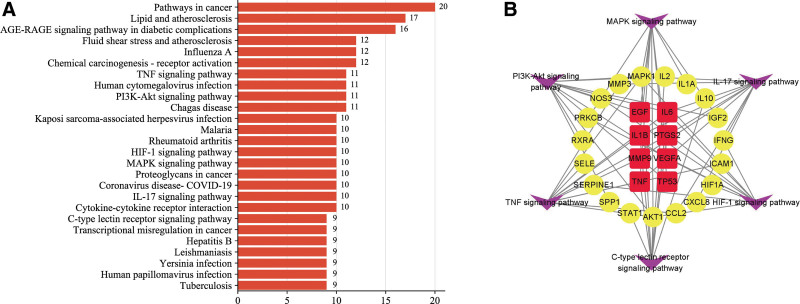
KEGG enrichment analysis and Target-Pathway network construction. (A) Details of the top 26 pathways in KEGG database. (B) Network between 6 key pathways and 27 therapeutic targets (inner ring represents 8 hub targets, middle ring represents 19 common targets, outer ring represents 6 KEGG pathways). KEGG = Kyoto Encyclopedia of Genes and Genomes.

To further predict the potential signaling pathways involved in the anti-PMOP effects of Fructus Lycii, these 41 overlapping genes were used for KEGG enrichment analysis. According to the descending order of count number contained in each pathway, we listed the top 26 enriched pathways (Fig. 6A). Among these pathways, IL-17 signaling pathway, TNF signaling pathway, MAPK signaling pathway and C-type lectin receptors signaling pathway can regulate inflammation; PI3K-Akt signaling pathway and HIF signaling pathway were involved in the biological process of angiogenesis. Furthermore, a Target-Pathway network was conducted to present the therapeutic targets enriched in these 6 signaling pathways (Fig. 6B).

## 4. Discussion

PMOP has become a severe public health problem worldwide with the progress of the aging population.^[27]^ Due to the side effects of current anti-osteoporosis drugs, it is urgent to develop a more effective and safer treatment strategy.^[28]^ Lycii Fructus, a common plant fruit with properties of drug homologous food,^[10]^ has been widely used to treat kidney deficiency-induced bone diseases. Also, many studies showed its bone protect effects in the OVX animal model.^[13,14]^ Our present study comprehensively explored the potential mechanisms of Lycii Fructus against PMOP through network pharmacology approach.

Through excavating and analyzing the target genes-related to Lycii Fructus and PMOP, we obtained 41 overlapping genes that were regarded as therapeutic targets of Lycii Fructus treating PMOP. The subsequent GO enrichment analysis on the 41 target genes revealed that inflammation and angiogenesis were 2 major biological processes involved in this anti-PMOP effects. According to the network of Herb-Ingredient-Target-Disease, 3 major indigents including quercetin, glycitein and stigmasterol were identified. By literature review, quercetin was found to alleviate OVX-induced osteoporosis through inhibiting inflammation^[29]^ and promoting angiogenesis.^[30]^ Both glycitein and stigmasterol had an obvious inhibition on inflammation, and effectively suppress osteoclast-caused bone resorption.^[31,32]^ Thus, considering our findings and literature, it could be concluded that these active ingredients are the important material foundation of Lycii Fructus against PMOP.

Based on the findings in KEGG enrichment analyses, there were 6 signaling pathway were associated with angiogenesis and inflammation. PPI topological screening with DC, BC and CC showed 13 hub targets that were VEGFA, TNF, IL-6, PTGS2, EGF, TP53, MMP9, IL1B, PPARG and ESR1. The Target-Pathway network showed that the therapeutic targets especially the 13 hub ones largely enriched in these 6 signaling pathways. Overall, we speculated that Lycii Fructus treats PMOP possibly through regulating of 2 biological processes and 6 signaling pathways.

### 4.1. Inflammation

Inflammation plays an important role in the development of PMOP,^[33]^ as massive evidence demonstrated that OVX-induced animals have systemic and chronic inflammation.^[34]^ Here, we identified TNF, IL-1B and IL-6 as the hub targets. All of them have shown close association with osteoclast bone resorption.^[35–37]^ TNF synthesized by T-lymphocytes can protect osteoclasts from apoptosis and prolong their lifespan.^[38]^ Both IL-1B and IL-6 can act on osteoclast lineage cells to stimulate osteoclastogenesis, leading to a high bone resorptive capacity.^[39,40]^ As the Target-Pathway network shown, these 3 hub targets were all enriched in IL-17 signaling pathway, TNF signaling pathway and MAPK signaling pathway. Literature review further demonstrated that these 3 pro-inflammatory cytokines are critical molecular targets in regulating the signal transduction of these pathways. In summary, anti-inflammation might be a potential mechanism of Lycii Fructus for PMOP treatment.

### 4.2. Angiogenesis

Bone formation requires an involvement of angiogenesis not only for it provides essential oxygen, nutrients and bone cells,^[41]^ but also for its directly angiogenesis-osteogenesis coupling.^[42,43]^ In contrast, reduction of angiogenesis genetically or pharmacologically is an important pathogenesis to many bone diseases, such as fracture ununion, femoral head necrosis and osteoporosis.^[44]^ VEGFA and EGF were 2 hub therapeutic targets of Lycii Fructus. Both of them can drive endothelial cell proliferation, vessel pruning and anastomosis for angiogenesis.^[45,46]^ VEGFA is transcribed and translated after HIF-1α binds to the promoter of VEGF gene, therefore VEGFA is a downstream target of HIF-1 pathway.^[47]^ EGF can activate PI3K-Akt pathway by binding to EGF receptor.^[48]^ As the Target-Pathway network shown, these 2 hub targets were all enriched in HIF-1 pathway and PI3K-Akt pathway. Thus, promotion of angiogenesis regulated by HIF-1 pathway and PI3K-Akt pathway might play an critical role in the anti-PMOP effects of Lycii Fructus.

Some limitations exist in the present study. Firstly, the active ingredients of Lycii Fructus were obtained from prediction of computer arithmetics, pharmacokinetic profile in the human body still needs to be detected. More importantly, potential therapeutic targets, biological processes and signaling pathways of Lycii Fructus were revealed through network pharmacology, while subsequent animal or cellular validations were not involved to verify its exact mechanisms.

## 5. Conclusions

Through network pharmacology, we systemically predict 41 therapeutic targets of Lycii Fructus. The identification of 2 major biological processes (inflammation and angiogenesis) and the key pathways (IL-17 pathway, TNF pathway, MAPK pathway, PI3K-Akt signaling pathway, and HIF signaling pathway) indicates that Lycii Fructus treats PMOP in a direct or indirect synergy way of multi-targets, muti-biological processes and multi-pathways. Although subsequent validations are still needed to determine the exact mechanism of Lycii Fructus, this study provide promising directions for future research.

## Author contributions

**Conceptualization:** Jianbo Wang, Dandan Mao, Jian Zhao.

**Data curation:** Jianbo Wang, Leyan Li.

**Formal analysis:** Leyan Li, Dandan Mao, Hongkan Lou, Jian Zhao.

**Funding acquisition:** Dandan Mao, Hongkan Lou.

**Investigation:** Yi Wang.

**Methodology:** Leyan Li, Shuiqi Cai, Jian Zhao.

**Project administration:** Shuiqi Cai.

**Software:** Jianbo Wang, Yi Wang, Leyan Li, Shuiqi Cai, Dandan Mao, Jian Zhao.

**Supervision:** Jianbo Wang, Hongkan Lou.

**Validation:** Jianbo Wang, Hongkan Lou.

**Visualization:** Yi Wang, Dandan Mao, Hongkan Lou.

**Writing – original draft:** Jianbo Wang, Jian Zhao.

**Writing – review & editing:** Jianbo Wang, Jian Zhao.
